# Enhancing the transduction efficiency of lentiviral vectors in CAR-T cell therapy through an optimization workflow

**DOI:** 10.3389/fmed.2026.1727427

**Published:** 2026-03-12

**Authors:** Rita Ferreira, Jaciara Fernanda Gomes Gama, Ana Godinho-Santos, Joao Goncalves

**Affiliations:** Molecular Microbiology and Biotechnology Laboratory, Research Institute for Medicines (iMed.ULisboa), Faculdade de Farmácia da Universidade de Lisboa, Lisbon, Portugal

**Keywords:** CAR-T cell therapy, lentiviral vectors, transfection, transduction, Jurkat E6-1, PBMCs

## Abstract

Efficient lentiviral (LV) transduction is a cornerstone of CAR-T manufacturing, yet performance is often construct-specific and highly sensitive to production and delivery parameters. We developed a stepwise optimization workflow using an underperforming anti-FITC-CAR in Jurkat E6-1 cells and validated generalizability with a well-performing HER2 CAR (pHR_SFFv_4D5-WT-Highest), followed by translational testing in primary PBMCs. The strategy sequentially tuned LV concentration, brief agitation during transduction, packaging system, DNA input balance, and addition of a transduction enhancer, with outcomes quantified by flow cytometry (tdTomato and HA or c-myc tags). Concentrated supernatants and a short 2-h shaking step improved signal definition and yield; incorporating an alternative packaging plasmid and a modest DNA rebalance further increased performance. With a low-dose enhancer, Jurkat transduction with the anti-FITC-CAR arose from ∼1% to ∼40–50% tdTomato^+^HA^+^ cells (∼5–50-fold improvement, 96 h). The comparator HER2 construct—already efficient—also benefited, increasing from ∼76 to ∼88%, indicating the workflow’s utility even for high-baseline vectors. In PBMCs, the same conditions achieved ∼10% transduction at 96 h, consistent with the greater refractoriness of primary T cells and highlighting avenues for future gains via complementary steps. Overall, this modular, low-complexity optimization provides a reproducible template to rescue underperforming constructs and incrementally boost robust vectors, supporting more reliable lab-scale CAR-T engineering and offering a tractable starting point for primary T-cell protocols.

## 1 Introduction

Chimeric antigen receptor (CAR) T-cell therapy has transformed cancer immunotherapy by genetically modifying and enabling patient-derived T cells to selectively recognize and destroy target cells. This therapy utilizes chimeric antigen receptors (CARs), enabling them to target specific antigens present on different cells. Once infused back into the patient, CAR-T cells initiate a specific immune response against the target, leading to cell destruction (1, 2). Clinically approved products have achieved durable responses in several hematologic malignancies, including relapsed or refractory B-cell acute lymphoblastic leukemia, diffuse large B-cell lymphoma, and multiple myeloma (3, 4). There are currently several CAR-T cell therapies approved by FDA (Food and Drug Administration) and European Medicines Agency (EMA) targeting specific hematologic malignancies, including relapsed or refractory B-cell acute lymphoblastic leukemia (ALL) (5), diffuse large B-cell lymphoma (DLBCI) (6), mantle cell lymphoma (MCL) (7), and follicular lymphoma (FL) (8). These therapies have demonstrated significant clinical benefits, including high response rates and durable remissions in patients with otherwise limited treatment options.

Ongoing research aims to expand the use of CAR-T cell therapy beyond cancer including solid tumors (9), autoimmune diseases (10), infection diseases (11, 12), with early studies demonstrating encouraging results in diseases such as systemic lupus erythematosus (SLE) (13) and pemphigus vulgaris (10, 13, 14). In fact, as of 2024, over 1,000 CAR-T clinical trials are registered on ClinicalTrials.gov, with over 75% conducted in the United States and China (15). However, despite the first-in-human CAR-T cell infusion occurring over 20 years ago, most of these trials remain in early-phase development (phases I and II) (16, 17), particularly for solid tumors like triple-negative breast cancer (TNBC), where tumor infiltration, antigen escape, and microenvironmental barriers remain key limitations (14, 18).

Overcoming current limitations in CAR-T cell therapy requires innovative strategies; nonetheless, all CAR-T cell therapies fundamentally rely on the synthesis of novel CAR constructs, efficient transduction and CAR surface expression (19, 20). At the manufacturing level, all CAR-T therapies depend on robust genetic modification of T cells to achieve high, stable CAR expression without compromising cell viability or phenotype. Currently, two main approaches are employed for gene delivery into human cells: non-viral systems and viral systems. Non-viral methods, including electroporation and lipid nanoparticles (21), are generally considered safer due to lower risk of insertional mutagenesis and immunogenicity; however, they often suffer from low efficiency and transient gene expression. In contrast, viral vectors (22), such as lentiviruses (LV) and retroviruses (RV), provide high transduction efficiency and stable genomic integration, despite persistent challenges such as limited packaging capacity, production scalability, potential genotoxicity, and high costs. Optimization is particularly important when working with underperforming constructs that yield insufficient CAR expression for functional studies or preclinical validation. In such cases, incremental modifications to production and delivery steps may rescue performance without resorting to redesigning the CAR itself. Addressing these challenges, key factors must be optimized, including the choice of producer cell line, transfection conditions, and viral particle stability (19, 22). The selection of producer cells is especially critical, as it affects viral yield, infectivity, and the presence of undesirable by-products (23, 24). A prevalent and efficient method for generating lentiviral particles (LVs) involves the transient transfection of Human Embryonic Kidney (HEK) 293T cells using plasmids encoding the transfer cassette, packaging viral proteins (gag/pol), and envelope proteins. HEK 293T cells are preferred for their high transfectability and consistent virus output (25, 26). However, alternative cell lines such as HEK 293ET (27), HEK 293FT (25), HeLa (28), and HT1080 (29) have also been employed efficiently depending on the vector type and application.

While enhancements such as vector concentration, altered packaging plasmids, or use of chemical enhancers are described individually in the literature, their combined, sequential impact in a reproducible workflow has not been thoroughly examined in the context of CAR-T cell generation (30). Furthermore, comprehensive optimization of culture conditions, including the choice of serum composition that yields higher transfection efficiency, as well as inoculation density and incubation parameters, will profoundly influence the overall quality and quantity of retroviral vectors in gene therapy applications (23). In fact, an optimized CAR-T cell manufacturing process may not be universally applicable to all CAR constructs, and each stage—from viral vector production to CAR expression—could benefit from further refinement and optimization. Nevertheless, specific guidelines regarding optimization for increasing viral production and viral transduction efficiency are not available.

In this study, we present a structured optimization strategy to improve LV-mediated delivery of a model anti-FITC-CAR that initially exhibited poor transduction efficiency in Jurkat E6-1 cells and peripheral blood mononuclear cells (PBMCs). We systematically evaluated the effect of viral concentration, brief agitation during transduction, packaging plasmid selection, plasmid DNA ratio adjustment, and the addition of a commercial transduction enhancer. This optimization of lentiviral transduction for expression of a control CAR recognizing FITC (anti-FITC-CAR) in a Jurkat E6-1 cell line demonstrated how incremental adjustments across production, concentration, and transduction steps led to significant gains in transduction efficiency. The optimized conditions were validated using a high-efficiency HER2 CAR construct and tested in peripheral blood mononuclear cells (PBMCs) to assess translational relevance. This work provides a practical, modular framework for improving LV transduction efficiency in both research and preclinical CAR-T applications.

## 2 Material and equipment

### 2.1 Material

Ampicillin

Bovine Serum Albumin (BSA)

Cell Culture Flasks

Cell lines Jurkat E6-1 and HEK 293ET

Culture plates

Dulbecco’s Modified Eagle Medium (DMEM)

*E. coli* - One Shot™ Stbl3™

Fetal Bovine Serum (FBS)

Ficoll-Paque PLUS

Fixation Buffer

Human Interleukin-2 Recombinant Protein

LentiBlast

Lenti-X™ Concentrator

Lenti-X™ p24 Rapid Titer Kit (Single Wash)

LIVE/DEAD™ Fixable Near-IR Dead Cell Stain Kit

Luria Broth (LB) medium

Micropipettes and Tips

Monoclonal antibodies (α-c-myc tag clone 9E10; α-HA tag clone 16B12)

NucleoBond Xtra Midi

Opti-MEM medium| Reduced Serum Medium

Penicillin-Streptomycin Amphotericin B Solution (PSA)

Peripheral Blood Mononuclear Cells (PBMCs)

Phosphate-buffered saline (PBS)

Pipettes

Plasmid DNAs

Polyethylenimine (PEI)

RPMI 1640 medium

Tubes (1.5, 15 and 50 mL)

### 2.2 Equipment

Centrifuge

CO_2_ incubator

Cytek^®^ Aurora cytometer

Hemacytometer

Inverted optical microscope

Laminar flow hood

Thermomixer

Varioskan™ LUX

Vortex

Water bath (37°C)

Centrifuge tubes (1.5, 15 and 50 mL)

### 2.3 Plasmids and cells

Five different plasmids were used in the following assays: a transfer plasmid containing our target insert flanked by long terminal repeat (LTR) sequences (anti-FITC-CAR, VectorBuilder or pHR_SFFv_4D5-WT-Highest-CAR_RHL004, also named WT-CAR Plasmid #164826, Addgene); a packaging plasmid encoding essential viral genes—psPAX2 also PAX2, Plasmid #12259, Addgene or pCMVR8.74 from now on mentioned as R8.74, Plasmid #22036, Addgene); and an envelope plasmid encoding the VSV-G envelope protein (pMD2.G, Plasmid #12259, Addgene).

Anti-FITC-CAR is 10371 base pairs (bp) long and contains a modified single-chain variable fragment (scFv) to recognize the fluorescent protein named fluorescein isothiocyanate (FITC) under EF1α promotor. This in-house designed construct has fluorescent protein (tdTomato) as an expression reporter with an HA tag for the detection of CAR surface expression. WT-CAR is 10546 bp long with a CAR to recognize Human Epithelial Receptor-2 (HER2) and its expression under the control of SFFV promotor can be detected by the c-myc tag (31). Both CAR constructs are second generation containing the 4-1BB costimulatory domain and the CD3ζ activating domain and are represented in Supplementary Figure 1.

HEK 293ET cell line is an embryonic cell line that exhibits epithelial morphology and adherence. It was used for lentiviral production because of its high susceptibility to transfection (a kind gift from Chengyu Jiang, Peking Union Medical College). This cell line is derived from HEK 293 cells by transformation with both simian virus 40 (SV40) large T antigen and Epstein-Barr nuclear antigen (EBNA) 1 (32). Human T-Cell Lymphoma Jurkat cell line E6-1 was used as a model for T cell transduction (NIH HIV Reagent Program, Catalog ARP-177). PBMCs from healthy donors were isolated from buffy coats provided by Instituto Português do Sangue e da Transplantação (IPST, Lisbon) after obtaining written informed consent and following the approval of the Ethics Committee for Human Research (CEISH) of Faculdade de Farmácia da Universidade de Lisboa.

## 3 Methods

All methods are thoroughly described below, and the workflow is illustrated in Supplementary Figures 2, 3.

### 3.1 DNA preparation

*E. coli* bacteria (Thermo Scientific) transformed with each plasmid were inoculated in 5 mL LB medium containing the appropriate antibiotic (ampicillin 100 μg/mL). After overnight incubation at 30°C with vigorous shaking (220 rpm), pre-inoculum growth was performed. Then, an inoculum was prepared by diluting the overnight culture at 1:1,000 and maintaining it at 30°C with vigorous agitation (220 rpm) to ensure proper aeration for up to 16 h. Plasmid DNA extraction was performed using NucleoBond Xtra Midi (Macherey-Nagel) according to the manufacturer’s instructions, and DNA quantification was conducted using Varioskan™ LUX (Thermo Scientific).

### 3.2 Cell culture

HEK 293ET cells were cultured in complete DMEM medium [DMEM supplemented with 10% FBS (Gibco) and 1% PSA (1,000 IU/mL penicillin G, 10,000 μg/mL streptomycin, and 25 μg/mL Amphotericin B, HyClone)]. Cells were maintained in T-75 flasks and enzymatically dissociated with trypsin at approximately 80–90% confluent (twice a week).

Jurkat E6-1 cells were cultured in complete RPMI 1640 medium (RPMI 1640 supplemented with 10% FBS and 1% PSA). This cell line grows in suspension with occasional clustering, and cell passage was performed every 2–3 days, following the medium characteristics, cell behavior, and density.

PBMCs were initially isolated from buffy coats provided by IPST using a Ficoll-Paque density gradient protocol and aliquoted based on the number of cells obtained and experimental requirements. For each assay, PBMCs were thawed and resuspended in complete RPMI 1640 supplemented with IL-2 (100 IU/mL; PeproTech) to reach a concentration of 1.5 × 10^6^ cells/mL just before adding TransAct read-to-use reagent for T cell activation (1:100 dilution; Miltenyi Biotec). The prepared cell suspension with TransAct was then seeded in 48-well plates (0.6 × 10^6^ cells/well) for 24 h of activation before transduction at 37°C and 5% CO_2_.

All cell cultures were meticulously maintained at 37°C in a humidified atmosphere (∼95% humidity) with 5% CO_2_ to ensure optimal conditions for cellular growth and lentiviral production.

### 3.3 Lentiviral vectors production

LVs generation involved transfection of HEK 293ET cells with three plasmids: VSV-G envelope (pMD2.G), packaging (PAX2 or R8.74), and transfer (anti-FITC-CAR or WT-CAR) plasmids.

Cells were seeded 1 day before transfection in a 6-well plate at 0.5 × 10^6^ cells/mL in a final volume of 2 mL/well. Plasmid proportions used for transfections were 0.5:3:1, 0.5:3:2, or 0.5:3:4 for envelope: packaging: transfer with 3 μg of total plasmid DNA/well. Plasmid DNAs and PEI were diluted in Opti-MEM (Gibco) medium to a concentration of 3 μg and 9 μg, respectively, each in 150 μL, according to the published protocol (33). The diluted PEI was then mixed with diluted DNA to a final volume of 300 μL and incubated at room temperature (RT) for 30 min. Subsequently, the PEI-DNA mixture was added slowly, drop-wise, to each well of cells, without mixing. The medium was changed 6 h post-transfection with fresh warm complete DMEM medium. All cultures were maintained at 37°C and 5% CO_2_ until viral particle collection 48 h post-transfection. At this point, LVs contained supernatants were either stored directly at –80°C or concentrated 10-fold using the Lenti-X™ Concentrator (Takara Bio), following the manufacturer’s instructions. All viral productions were stored at –80°C until the day of transduction. After transduction, quantification of viral supernatants was performed using Lenti-X p24 Rapid Titer Kit (Single Wash) (Takara Bio).

### 3.4 Transduction

For Jurkat E6-1 transduction, cells were counted to ensure that exponential phase of growth was reached, and cells were seeded at a density of 1 × 10^6^ cells/mL in a 24-well plate at a final volume of 500 μL (0.5 × 10^6^ cells/well). Transduction occurred immediately afterward by adding directly 50 μL or 150 μL of viral suspension into each well or incubated with cells in tubes while shaking for 2 h at 300 rpm at 37°C in a thermomixer (Labolan). To enhance transduction efficiency, LentiBlast Premium (OZBioscience) was used in same assays, following the manufacturer’s protocol. Briefly, 50 μL or 150 μL of viral suspension was placed into tubes and then LentiBlast was added to the tubes at 1 or 10% in both conditions (50 μL or 150 μL of LVs). The tubes were gently mixed by inversion. Then, the viral suspension-LentiBlast mixture was incubated with cells in tubes while shaking for 2 h at 300 rpm at 37°C in a thermomixer (Labolan). Following this incubation, the cells were transferred to 24-well plates and incubated at 37°C with 5% CO_2_ in a humidified incubator.

As mentioned before, PBMCs were seeded at a density of 1.5 × 10^6^ cells/mL in a 48-well plate (400 μL per well) and activated for 24 h prior to transduction. Transduction protocol was performed as previously described above, in 48-well plate format instead of 24-well plate.

Cell collection for flow cytometry was performed 48 h and 96 h post-transduction, as well as 7 days in some assays. During all collections, fresh medium was added to ensure optimal conditions for continuous cell growth and maintenance up to 7 days.

### 3.5 Flow cytometry

Transduction efficiency was determined by using flow cytometry in 96-well round-bottomed plates. For each condition, approximately 0.5 × 10^6^ cells from each well were collected. Cells were centrifuged at 450 × g for 5 min at RT, and the supernatant was discarded. Cells were then washed twice with 200 μL of PBS 1x to remove any remaining viral particles or debris, and then stained using LIVE/DEAD™ Fixable Near-IR Dead Cell Stain Kit (LD, Life Technologies) for 10 min at RT. After centrifugation to discard unbound LD, cells were stained with monoclonal antibodies against tags for CAR expression, such as α-c-myc (Alexa Fluor 647, BioLegend) and α-HA (Alexa Fluor 647, BioLegend), for WT-CAR and anti-FITC-CAR constructs, respectively. Transduction efficiency of anti-FITC-CAR in T cells was assessed using both tdTomato (fluorescence reporter protein) and HA tag expression. Immune profiling in PBMCs was also performed in parallel in some experiments using a panel of antibodies targeting pan-leukocytes (CD45), T cells (CD3, CD4, CD8), B cells (CD19), NK cells (CD56), and monocytes (CD14). Cells were incubated with antibodies for 30 min at RT protected from light. To remove unbound antibodies, cells were centrifuged at 450 × g for 5 min and washed twice with 200 μL of PBS containing 1% BSA and 0.04% azide (PBS/BSA/Az). Cells were fixed with fixation buffer (BioLegend) for 10 min at RT. Cells were centrifuged at 450 × g for 5 min and resuspended in 160 μL of PBS/BSA/Az. Acquisition was performed on a full-spectrum Cytek^®^ Aurora cytometer (Cytek^®^ Biosciences) and data was analyzed in FCS Express™ 7 software (*De Novo* software, version 7.18.0021).

### 3.6 Statistical analysis

Statistical analysis was performed with one-way analysis of variance (ANOVA), followed by *t*-test using GraphPad Prism version 10.1.2 (GraphPad Software). Differences between groups were considered statistically significant at *P* < 0.05.

### 3.7 Limitations

Optimizing a process can be a challenging task that requires careful consideration of many factors, such as the size of flasks and plates, selection of appropriate cell lines, cell density, time and maintenance of culture, and the proportion of viruses and cells. Additionally, centrifugation and washing must be performed with care to minimize the loss of cells. Other factors that might affect viral production and transduction efficiency, such as CAR construct redesign, DNA concentration, different envelope vectors, and different viral titers, were not evaluated. It is important to highlight that transduction enhancers other than LentiBlast were not included, as they were unavailable during the study period; nevertheless, they may offer additional methodological gains.

## 4 Results

**FIGURE 1:**
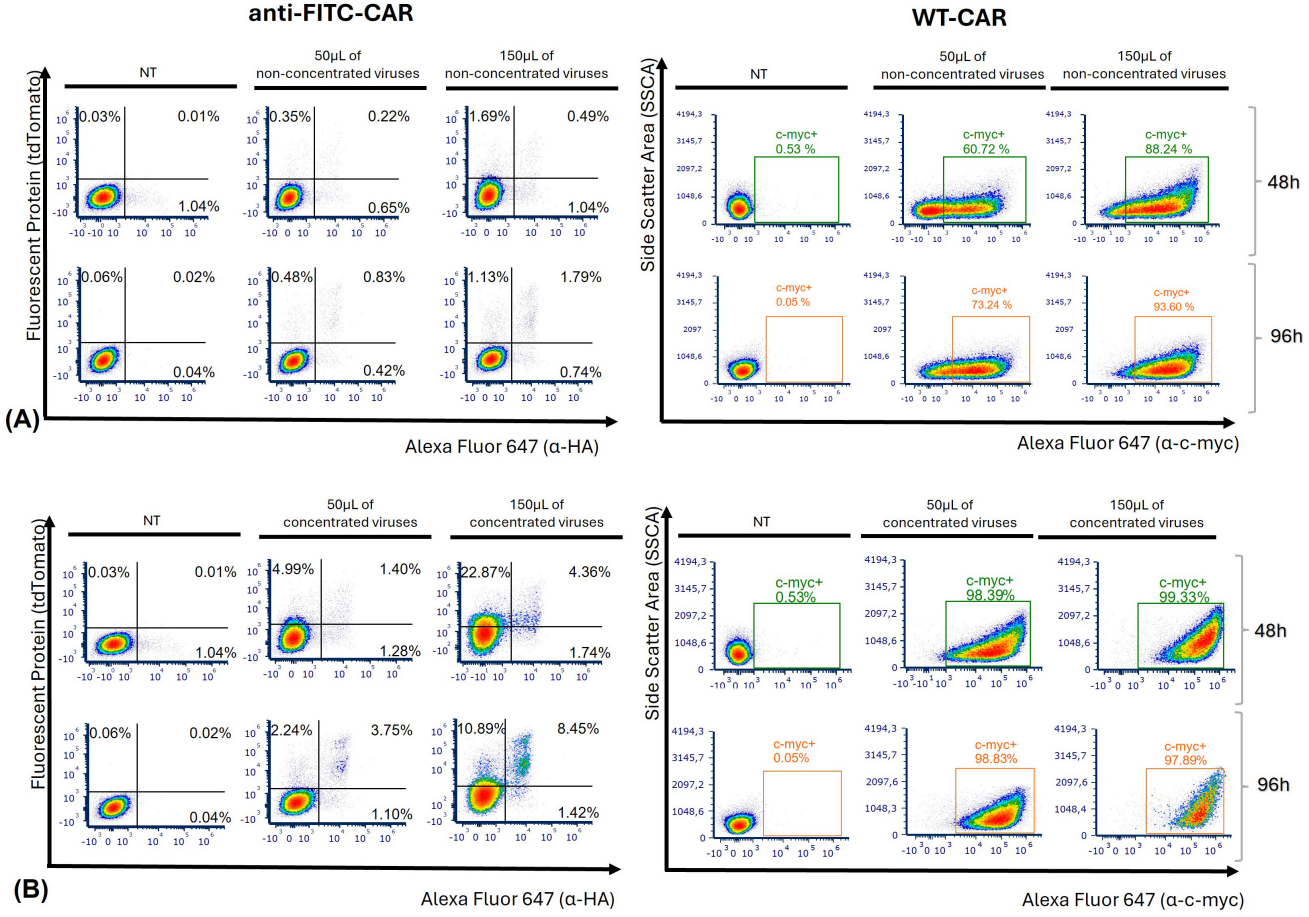
Transduction with new construct was confirmed with concentrated viruses. Jurkat E6-1 cells were transduced with 50 and 150 μL of anti-FITC-CAR or WT-CAR viral particles. Non-transduced (NT) cells were used as a negative control. Levels of tdTomato and HA tag or only c-myc tag were measured 48 and 96 h post-transduction to assess the percentage of transduced cells with anti-FITC-CAR or WT-CAR, respectively. The percentages of transduced cells with non-concentrated **(A)** or concentrated **(B)** LVs are shown in representative dot plots from 3 independent experiments.

To test the transduction efficiency of a new anti-FITC-CAR construct, which will serve as a reference for several CAR constructs, we first produced LVs by transfecting HEK 293ET cells with VSV-G envelope plasmid, PAX2 packaging plasmid, and anti-FITC-CAR construct, using a ratio of 0.5:3:1, as previously used for WT-CAR in our laboratory. LVs were first added directly to Jurkat E6-1 cells and transduction remained untouched for 48 h. Flow cytometry analysis employed a standard gating strategy across all samples to assess transduction efficiency using reporter tdTomato and HA tag for surface expression of CAR 48 and 96 h post-transduction (Supplementary Figure 4). The percentage of live cells expressing tdTomato and HA tag was less than 5% with the anti-FITC-CAR construct, regardless of the viral input (Figure 1A). For WT-CAR, 96 h post-transduction with non-concentrated particles yielded better results, reaching a mean of 47.7% of transduced cells with the lowest viral input (50 μL) and 76.2% with the highest viral input (150 μL) (Figure 1A). The high percentage of transduced cells using WT-CAR construct indicates that our initial protocol was efficient for this CAR construct. Nevertheless, it was not efficient for anti-FITC-CAR construct; therefore, further protocol refinement may be required to achieve adequate transduction levels. For this reason, we first concentrated viral supernatants containing anti-FITC-CAR LVs before transduction and obtained a significant increase in CAR expression. Using 150 μL of the virus, we observed a double positive population that expressed both tdTomato and HA-tag (mean of 8.3% at 96 h timepoint, Supplementary Figure 5A). We also observed an increase in the WT-CAR-concentrated LVs (mean of 96.1%, Supplementary Figure 5B, 2.0-fold change). As depicted in Figure 1, all experiments demonstrated superior outcomes when using concentrated viruses compared to non-concentrated ones for transduction with anti-FITC-CAR particles (4.68-fold change, *p* = 0.0011 for anti-FITC-CAR).

**FIGURE 2:**
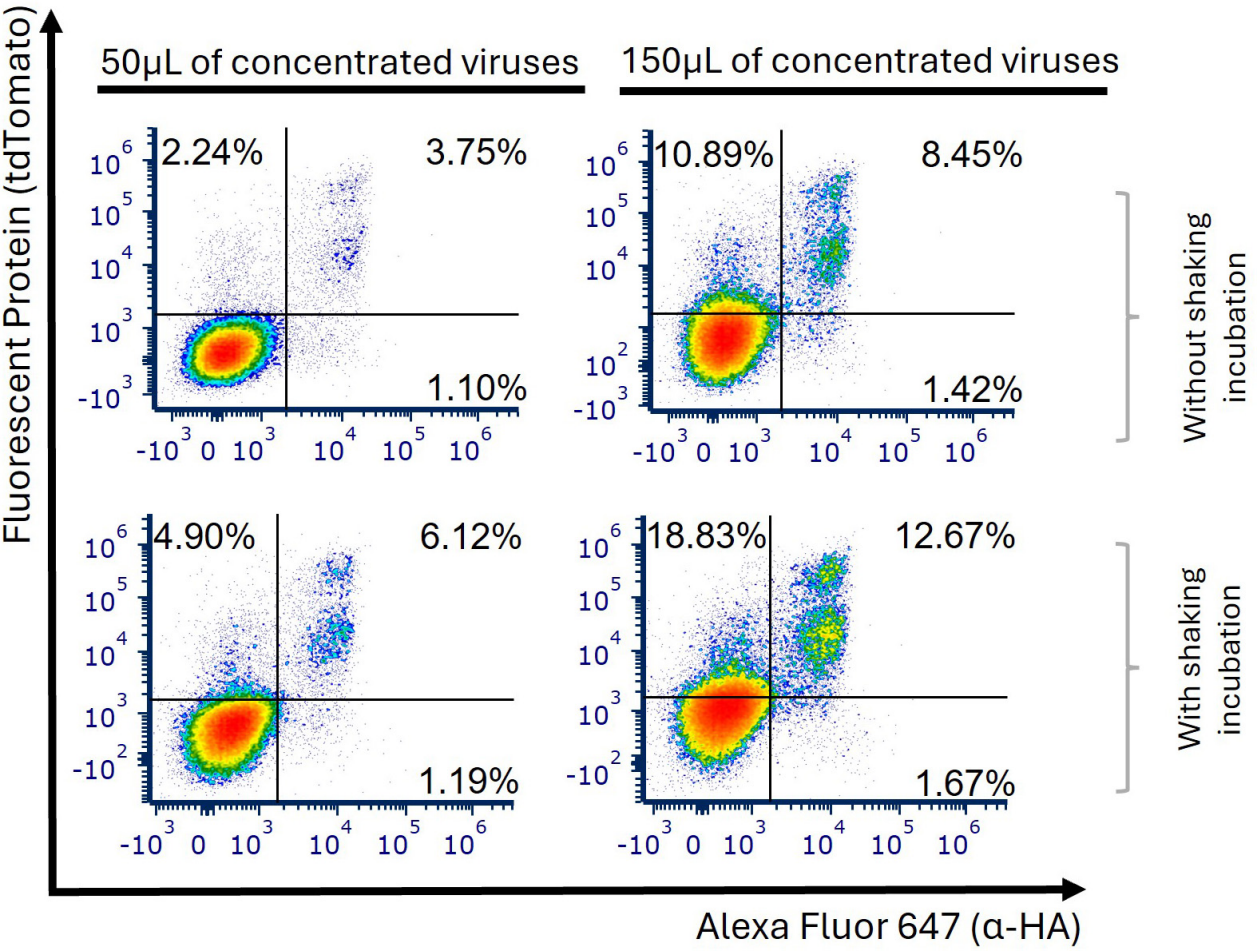
Transduction efficiency improved when conducted with shaking. Jurkat E6-1 cells were transduced using either 50 or 150 μL of concentrated anti-FITC-CAR particles. All samples were either subjected to shaking or left still for the initial 2 h post-transduction. Representative plots from 3 independent experiments, measured 96 h after transduction, display the levels of tdTomato and HA tag to evaluate the percentage of cells transduced with anti-FITC-CAR, both with and without shaking.

To improve the interaction between LVs and cells, concentrated viral particles were incubated with cells for 2 h at 37°C while being shaken at 300 rpm. We observed two more defined populations of double expression (tdTomato^+^HA^+^) at different levels (Figure 2), although the use of a shaker only showed a significant statistical improvement when 150 μL were used (1.41-fold change at 96 h post-transduction, *p* = 0.0133, Supplementary Figure 6A). Achieving a more precise definition of the tdTomato^+^HA^+^ population is crucial, particularly when conducting sort-purification. Consequently, we opted to maintain shaking conditions in the subsequent assays.

**FIGURE 3:**
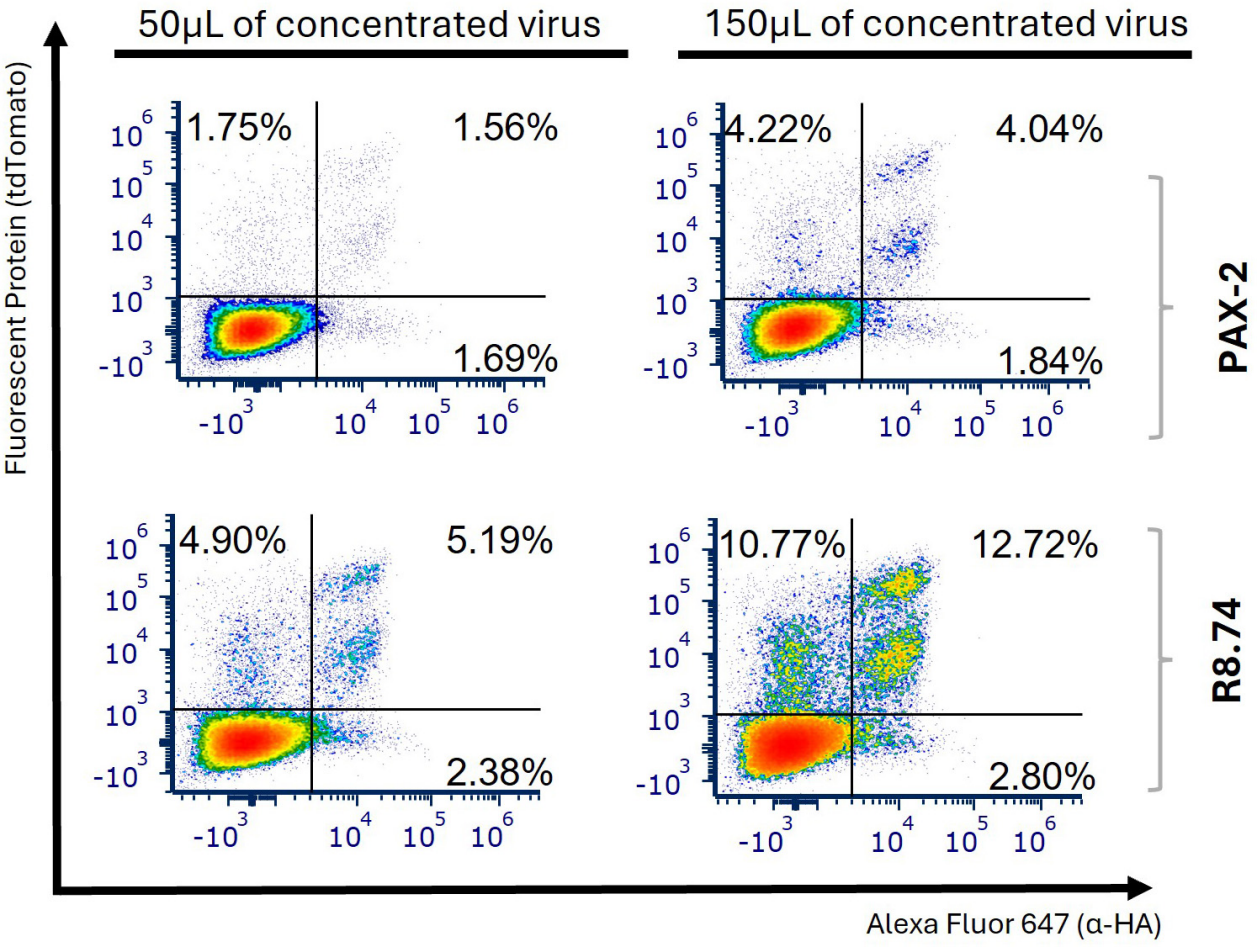
The efficiency of transduction was enhanced when the packaging plasmid R8.74 was utilized. Jurkat E6-1 cells were transduced with either 50 or 150 μL of concentrated anti-FITC-CAR viral particles, with agitation maintained for the initial 2 h post-transduction. Representative plots from 3 independent experiments illustrate the expression levels of tdTomato and HA tag 96 h following transduction, in order to evaluate the percentage of cells transduced with anti-FITC-CAR, employing various packaging plasmids during lentiviral vector production.

Another important factor affecting the production of LVs is the packaging plasmid used. The R8.74 packaging plasmid is increasingly recognized as a viable alternative to PAX2, known for its high efficiency and stability, and is becoming more prominent in Triple-Negative Breast Cancer research (34). Therefore, we decided to compare packaging plasmid R8.74 with previously used PAX2, when producing anti-FITC-CAR LVs. Although not statistically significant, we observed a tendency for higher efficiency of transduction using R8.74 (at 96 h post-transduction with 150 μL we observed a 2.17-fold change, Figure 3 and Supplementary Figure 7).

**FIGURE 4:**
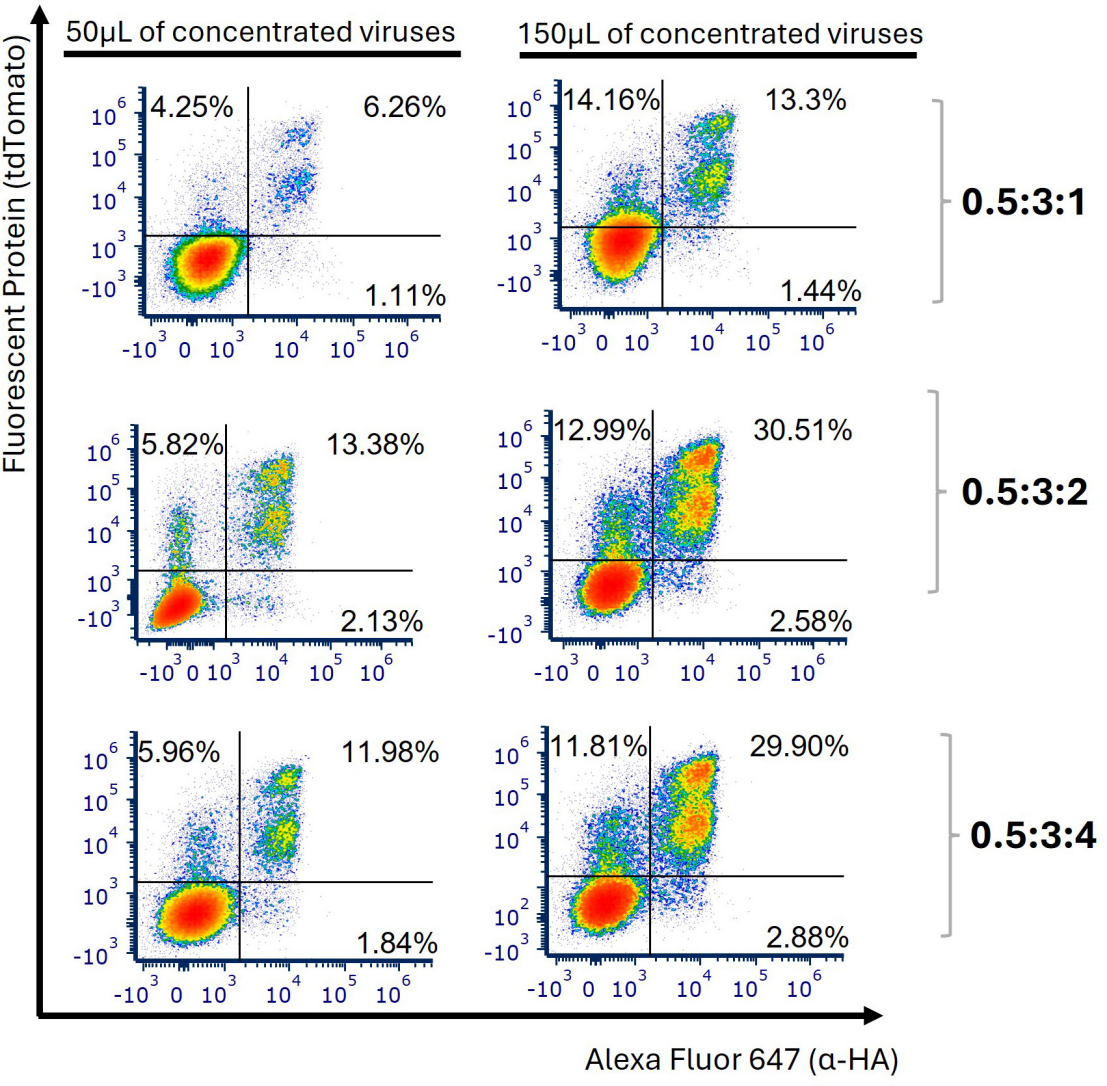
Transduction was optimized using a transfection ratio of 0.5:3:2. Jurkart E6-1 cells were transduced with 50 or 150 μL of concentrated anti-FITC-CAR particles, under shaking applied during the initial 2 h post-transduction. These particles were produced using R8.74 as the packaging plasmid. Representative plots from 3 independent experiments were generated to display the levels of tdTomato and HA tag, measured 96 h post-transduction, to evaluate the percentage of cells transduced with anti-FITC-CAR, using different transfection ratios for LVs productions.

To enhance transduction efficiency, we tested three different plasmid DNA ratios for the generation of LVs by increasing the amount of transfer plasmid while maintaining constant amounts of envelope plasmid (0.5) and packaging R8.74 plasmid (3). Utilizing 150 μL of LVs produced from the 0.5:3:2 ratio resulted in an increase in transduction efficiency, when compared to 0.5:3:1 and 0.5:3:4 ratios (mean of 25.9%, 1.40-fold change, Figure 4 and Supplementary Figure 8).

**FIGURE 5:**
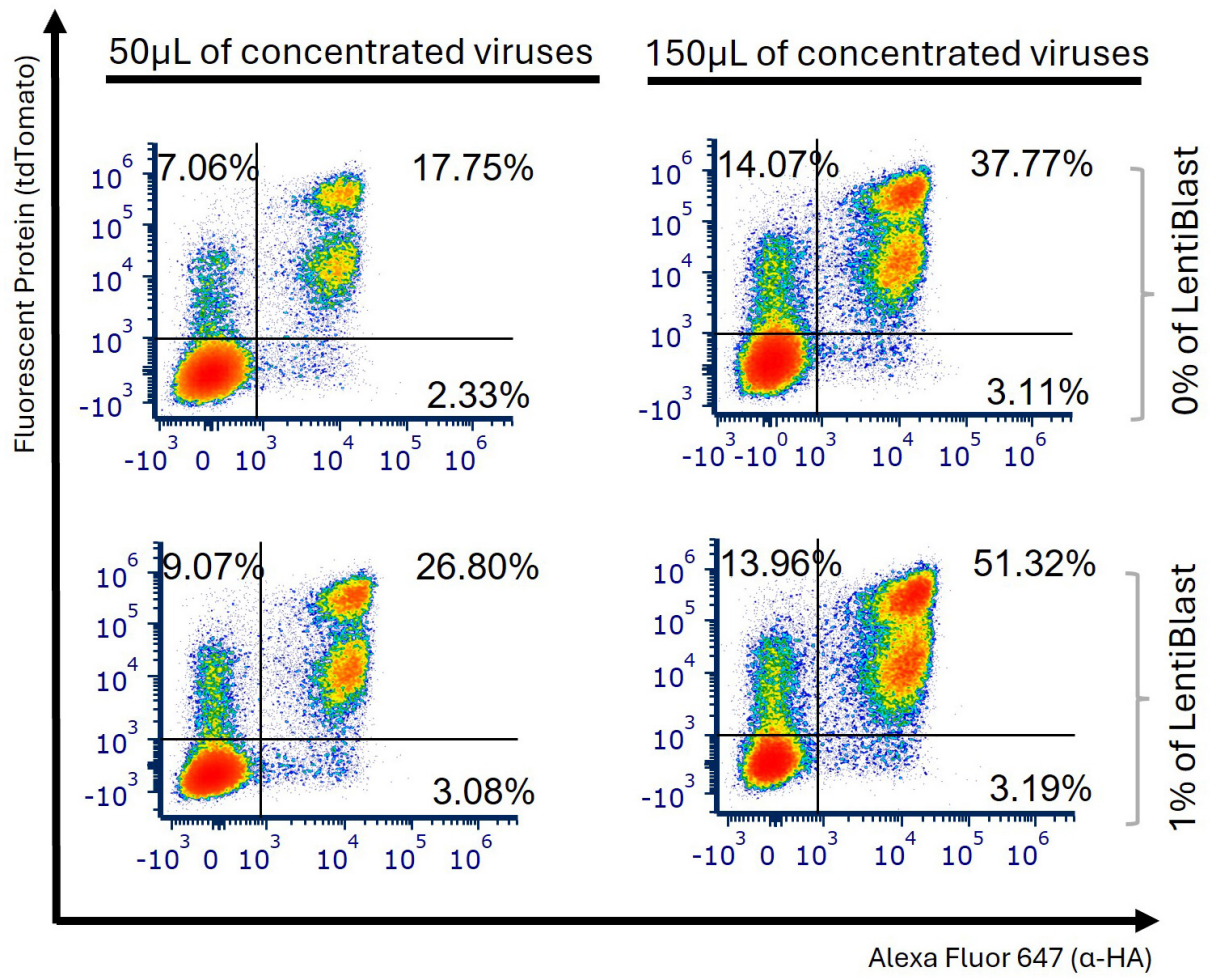
The efficiency of transduction was improved through the use of a transduction enhancer. Jurkat E6-1 cells were transduced with 50 and 150 μL of anti-FITC-CAR viral particles, with shaking applied during the initial 2 h post-transduction. These viral particles were produced with R8.74 as the packaging plasmid, with a transfection ratio of 0.5:3:2. Representative plots from 3 independent experiments illustrate the levels of tdTomato and HA tag measured 96 h post-transduction, which were used to evaluate the percentage of transduced cells with anti-FITC-CAR, employing LentiBlast.

We then attempted to integrate the improvements already made with the transduction enhancer LentiBlast Premium. Notably, the use of a reduced volume of LentiBlast, constituting 1% of the total viral volume (0.5 μL for 50 μL LVs or 1.5 μL for 150 μL LVs), proved effective across various viral quantities, ranging from approximately 5 to 16% (Figure 5 and Supplementary Figure 9B). The proportion of tdTomato^+^HA^+^ cells exceeds 35 and 50% at 96 h post-transduction with 50 μL and 150 μL of anti-FITC-CAR virus, respectively. Statistical analysis showed the significance of LentiBlast in enhancing transduction for anti-FITC-CAR in some conditions (at 96 h post-transduction with 150 μL we observed a 1.36-fold change, *p* = 0.0011, Figure 5 and Supplementary Figure 9B).

**FIGURE 6:**
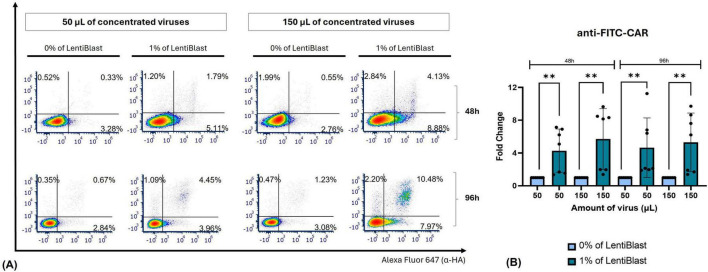
Transduction of primary cells with anti-FITC-CAR was accomplished using our optimized protocol. PBMCs cells were transduced with 50 and 150 μL of anti-FITC-CAR concentrated viral particles, which were produced using R8.74 as the packaging plasmid with a transfection ratio of 0.5:3:2. The transduction process was conducted under shaking conditions for the initial 2 h, with or without LentiBlast. The levels of tdTomato and HA tag were measured 48 and 96 h post-transduction to assess the percentage of transduced cells. **(A)** Representative plots from 7 independent experiments. **(B)** Plot summarizing fold-change differences. Data shown as mean ± SD (*n* = 5). ** *p* ≤ 0.01.

**FIGURE 7:**
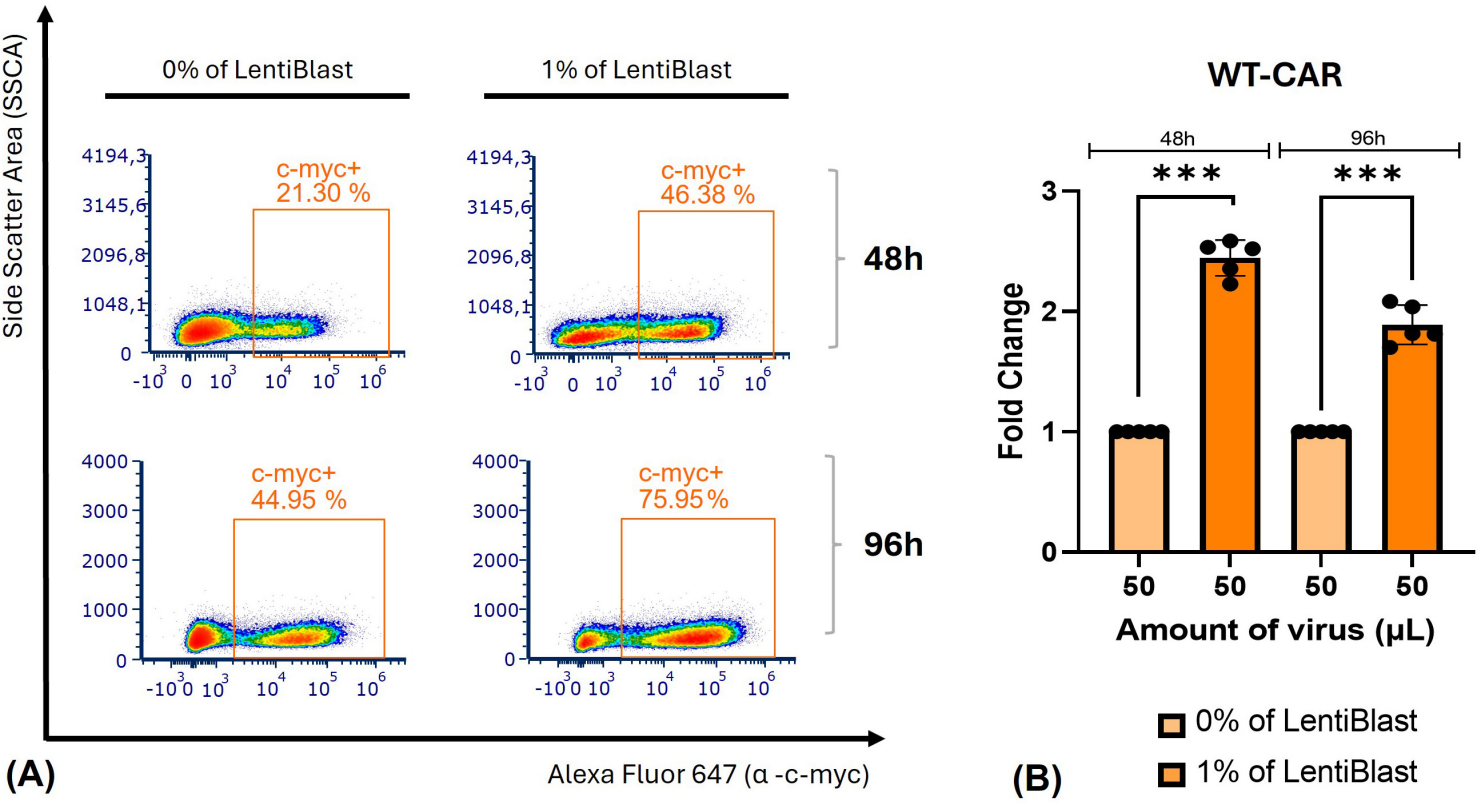
Transduction of primary cells with WT-CAR was enhanced by the optimized protocol. PBMCs were transduced using 50 and 150 μL of non-concentrated WT-CAR viral particles, under shaking applied during the first 2 h of transduction, with or without LentiBlast. The viral particles were produced using R8.74 as the packaging plasmid, with a transfection ratio of 0.5:3:2. The levels of the c-myc tag were assessed at 48 and 96 h post-transduction to determine the percentage of transduced cells with anti-WT-CAR. **(A)** Representative plots from 5 independent experiments. **(B)** Plot summarizing the fold-change differences. Data are presented as mean ± SD (*n* = 5). ***p ≤ 0.001.

Our transduction efficiency has markedly improved, increasing from an average of 1% to an impressive average of 50% (mean at 96 h, Supplementary Figure 9B). This enhancement was achieved in a T cell line by using 150 μL of concentrated LVs produced with R8.74 packaging plasmid in a 0.5:3:2 ratio, incorporating 1% of LentiBlast, and subjecting the mixture to shaking for a duration of 2 h. For clinical applicability, it is crucial to assess the impact of the same LVs in PBMCs. Transduction of PBMCs demonstrates reduced expression levels compared to Jurkat E6-1 cells, as illustrated in Figures 6, 7. Under the optimal transduction conditions identified in Jurkat E6-1 cells, we achieved approximately 10% transduction with 150 μL of virus and 1% LentiBlast after 96 hours (Figure 6A and Supplementary Figure 10). Of note, transduction levels were maintained until day 7 post-transduction (Supplementary Figure 11). A similar pattern of lower transduction efficiency was observed with WT-CAR, albeit with higher expression levels (Figure 7A). The consistency of these results was confirmed across donors, even with modest, yet expected, variability in major immune cell populations in primary samples (Supplementary Figure 12). Statistical analyses robustly support the hypothesis that LentiBlast significantly enhances transduction efficiency in most cases (Figures 6B, 7B).

## 5 Discussion

The efficacy of CAR-T cell therapies relies on the successful transduction of CAR constructs and their stable expression on the cell surface, which are crucial parameters for the therapeutic effectiveness of the engineered T cells. Therefore, enhancing the generation process of CAR-T cells is of paramount importance, and may depend on several factors including the CAR design itself, the vector system, and the transduction conditions. In this study, we investigated and optimized various parameters influencing lentiviral transduction efficiency, achieving significant improvements that were consistently validated across independent experiments. These enhancements were confirmed through flow cytometry, facilitating clear and reproducible monitoring of CAR expression on the cell surface.

One of the major determinants of success was the use of concentrated LVs, which markedly improved transduction rates compared to non-concentrated virus preparations (Figure 1). This approach effectively mitigates common challenges such as low viral titers or volume limitations. These findings align with studies indicating that concentration methods such as filtration and membrane-based concentration methods can yield functional titers as high as 6 × 10^9^ TU/mL, thereby facilitating more efficient gene delivery compared to unconcentrated or ultracentrifuged preparations (35, 36).

Previous research utilizing HIV models has indicated that viral particles produced by T lymphocytes under shaking conditions may experience a reduction in infectivity due to the shedding of envelope glycoproteins (37). In our study, agitation during the transduction resulted in enhanced expression of anti-FITC-CAR constructs using concentrated LVs (Figure 2). This apparent discrepancy may stem from fundamental differences in the viral lifecycle stage being targeted, as it demonstrates inefficient HIV-1 replication. Moreover, we used non-replicative LVs in our system which will not have an impact on viral production neither on cell-to-cell transfer. In fact, shaking during transduction contributed to better discrimination of positive versus negative populations, which is advantageous for downstream analysis and potential cell sorting procedures.

An additional aspect assessed in our study was the packaging plasmid system used for LV production. Although our results did not show a significant difference in transduction efficiency between psPAX-2 and R8.74 packaging plasmids when applied to our CAR construct, an increase in efficiency was nonetheless observed with the R8.74 plasmid (Figure 3). This trend aligns with previous studies indicating that R8.74 can surpass psPAX2 in performance in specific cell types, such as cardiac-derived c-kit^+^ cells (38). This underscores the necessity of considering the interaction between vector components and target cells must be considered on a case-by-case basis.

A significant finding in our optimization strategy was the identification of LentiBlast as a highly effective transduction enhancer. Its application led to substantial improvements in CAR expression in both Jurkat E6-1 cells and PBMCs (Figure 5), reinforcing the relevance of enhancer selection for effective gene delivery (39). To our knowledge, this is the first report of LentiBlast being utilized in CAR-T cell generation, and our results suggest that it holds potential as a straightforward and non-toxic enhancer compatible with both primary and immortalized T cells. In comparison to commonly used enhancers such as Retronectin, polybrene, and dasatinib, LentiBlast offered several operational advantages. Retronectin, although effective, with reported transduction rates exceeding 60% in primary T cells, requires pre-coating and centrifugation steps, increasing complexity (20). Polybrene can be cytotoxic at higher doses, while dasatinib—despite its recent success in enhancing transduction while preserving T cell phenotype—requires precise titration and is pharmacologically active (40). LentiBlast, in contrast, provided a balanced combination of efficiency and simplicity, requiring no additional equipment or cell surface manipulation.

Despite optimization efforts, the transduction efficiency in PBMCs remained significantly lower than that observed in Jurkat E6-1 cells. This finding aligns with prior evidence that primary T cells are inherently more refractory to lentiviral transduction due to inter-donor variability, heterogeneous activation states, differential receptor expression, and intracellular processing mechanisms (20, 38, 41). Although our efficiency in PBMCs did not surpass the clinically desirable threshold of 30%, these findings remain relevant, particularly at the preclinical or exploratory stage. It is important to note that achieving transduction efficiencies greater than 30% in primary T cells typically necessitates the use of advanced enhancers or multi-step protocols, often in conjunction with magnetic selection or expansion of transduced populations (42, 43). In this context, our data demonstrate a clear trend toward improvement and lay the groundwork for future refinement using complementary strategies such as plasmid concentration and purity, coating plates, other transfection reagents, or even exploring newer compounds such as BX795 or Vectofusin-1 (44–46).

In conclusion, this study elucidates the multifactorial nature of lentiviral transduction and underscores the need for comprehensive optimization of vector production, concentration methods, transduction conditions, and enhancer selection (47). Although several parameters, particularly those pertaining to primary cells, require further optimization, our findings offer valuable insights for both research and translational settings. The optimized protocol developed herein yielded robust and reproducible CAR expression in Jurkat E6-1 cells and provided a promising starting point for primary T cell engineering. Moreover, our optimized protocol improved transduction levels for WT-CAR construct, despite its higher baseline efficiency, strengthening its use as a platform useful for different CAR constructs. These results highlight the importance of methodical experimental design and the potential of simple yet effective modifications to substantially enhance the efficiency and reproducibility of CAR-T cell production.

## Data Availability

The original contributions presented in this study are included in this article/Supplementary material, further inquiries can be directed to the corresponding author.
