# Supplementary material for: Enhancing the transduction efficiency of lentiviral vectors in CAR-T cell therapy through an optimization workflow
Source: Front Med (Lausanne). 2026 Mar 12;13:1727427. doi: 10.3389/fmed.2026.1727427 (PMC13018915; doi:10.3389/fmed.2026.1727427)
Supplement: Supplementary file 1 [file Data_Sheet_1.pdf]

Supplementary Material

Supplementary Figures

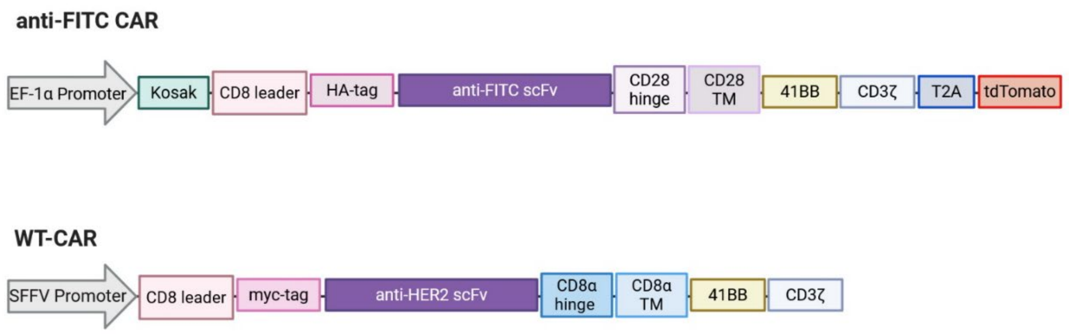

Supplementary Figure 1: Schematic diagram of anti-FITC-CAR and WT-CAR constructs. Both plasmids share CD8 leader sequence and identical intracellular signaling domain consisting of 4-1BB and CD3ζ. WT-CAR contains anti-human 4D5 scFv, a CD8 hinge and transmembrane domain and a myc-tag (bottom). In contrast, the anti-FITC CAR includes an anti-FITC scFv, a CD28 hinge and transmembrane domain, a Kosak sequence, and a tdTomato reporter and HA-tag (top).TM: transmembrane domain; scFv: single-chain Fragment variable. Created with BioRender.com.

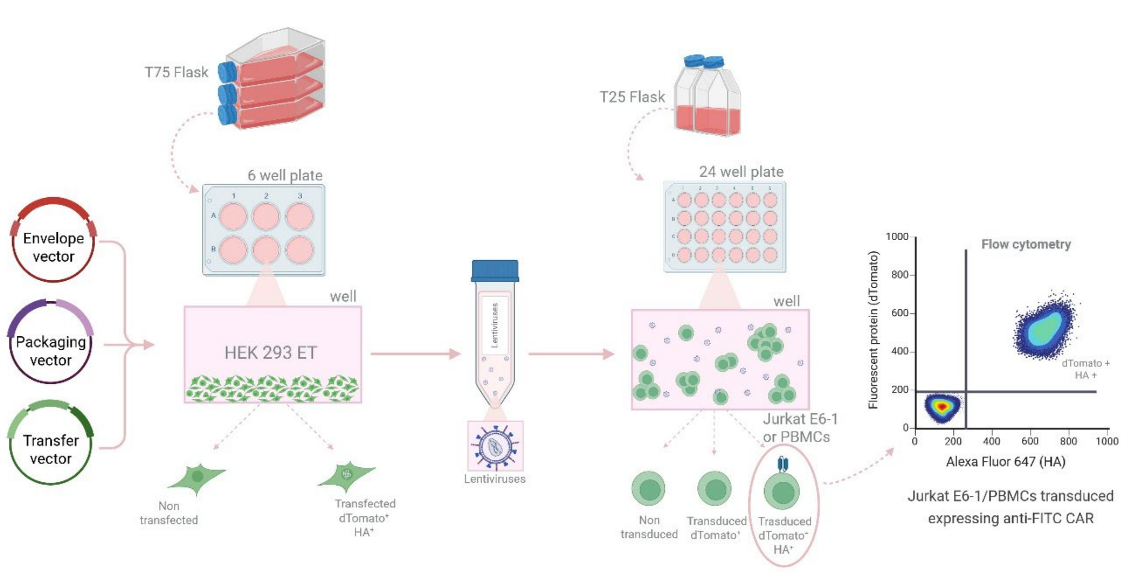

Supplementary Figure 2: Workflow protocol - Schematic representation. LV production by transfection in HEK 293ET, followed by transduction of Jurkat E6-1 cells or PBMCs. Flow cytometry was used to determine transduction efficiency levels. Cells were harvested at 70% confluence in a T75 flask and seeded in a 6-well plate (0.5×10^6/mL). Lentiviruses were harvested to be stored directly (non-concentrated) or to undergo a concentration protocol (concentrated). Subsequently, Jurkat E6-1

cells or PBMCs were seeded in a 24-well plate and transduced using either concentrated or non-concentrated viral supernatants. Flow cytometry was used to confirm transduction efficiency. Created with BioRender.com.

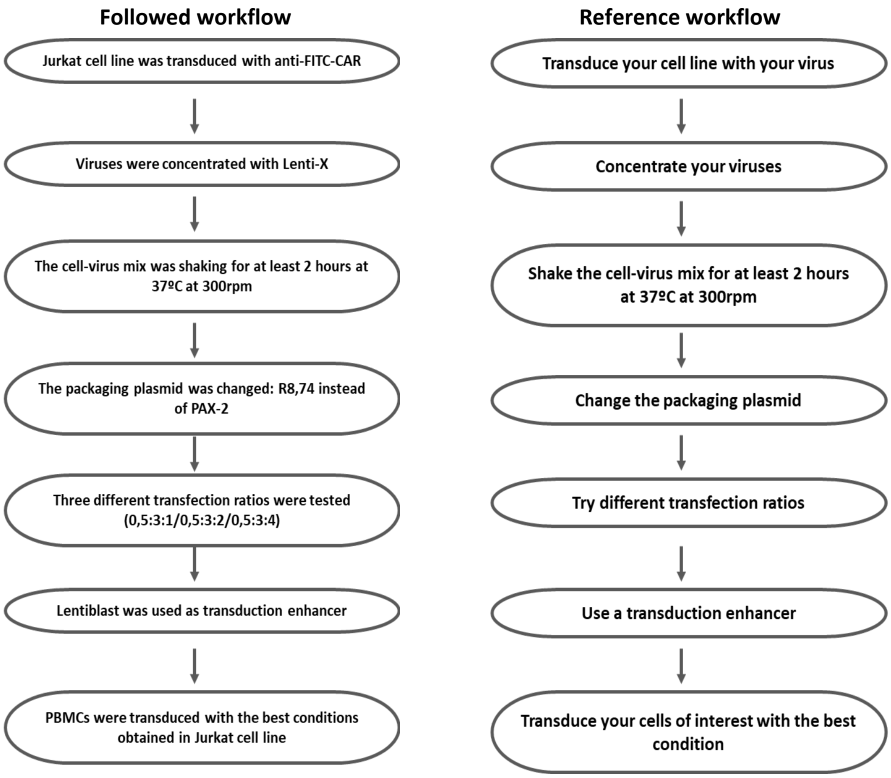

Supplementary Figure 3: Schematic representation of workflow improvements. Jurkat E6-1 cell line was initially transduced. However, because of the observed transduction impairments, several approaches have been implemented. These included the use of an LVs concentrator, a thermoshaker to enhance the effectiveness of LVs, comparison of two different packaging plasmids, comparison of different transfection ratios, and the use of a transduction enhancer. Finally, optimal conditions were established for the transduction of PBMCs.

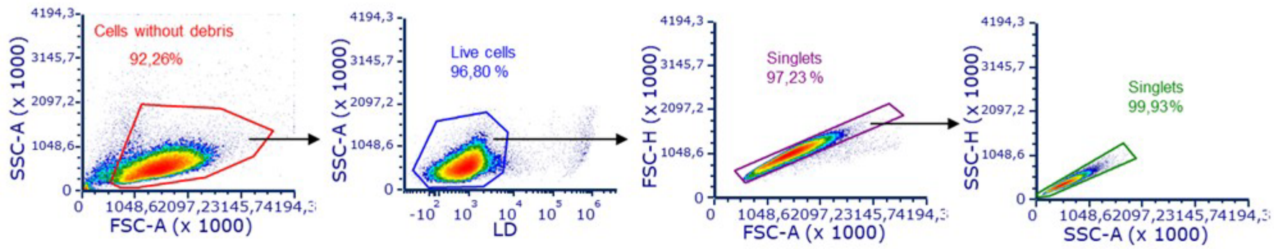

Supplementary Figure 4: Gating strategy used to address transduction efficiency in live single Jurkat E-61 cells or live single PBMCs. Gate on cells was applied to remove debris using SSC-A × FSC-A parameters, and live cells were selected to exclude non-viable cells. To analyze only single cells, two sequential gates were performed in plots FSC-A × FSC-H, followed by SSC-A × SSC-H.

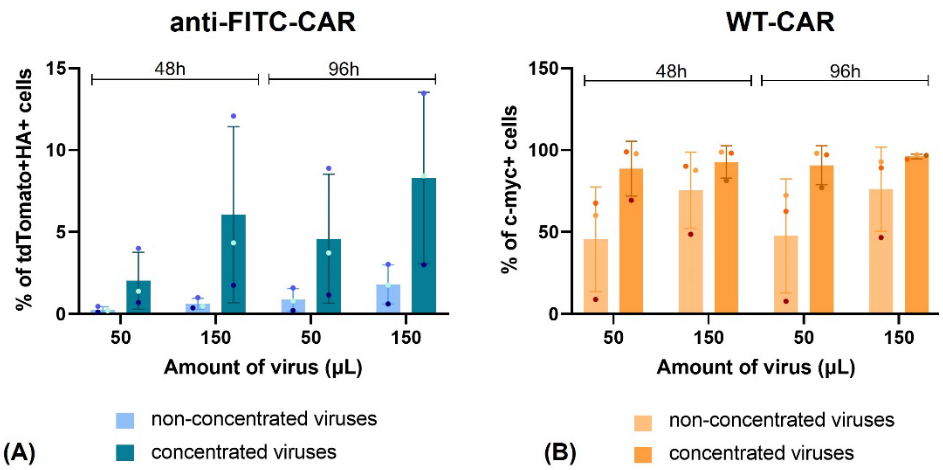

Supplementary Figure 5: Concentrated LVs improved transduction. Jurkat E6-1 cells were transduced with 50µL and 150µL of anti-FITC-CAR or WT-CAR LV particles. Non-transduced (NT) cells were used as negative controls. The levels of tdTomato and HA tag (A) or c-myc tag (B) were measured 48h and 96h post-transduction to assess the percentage of transduced cells with anti-FITC-CAR or WT-CAR, respectively; data shown as mean ± SD (n=3).

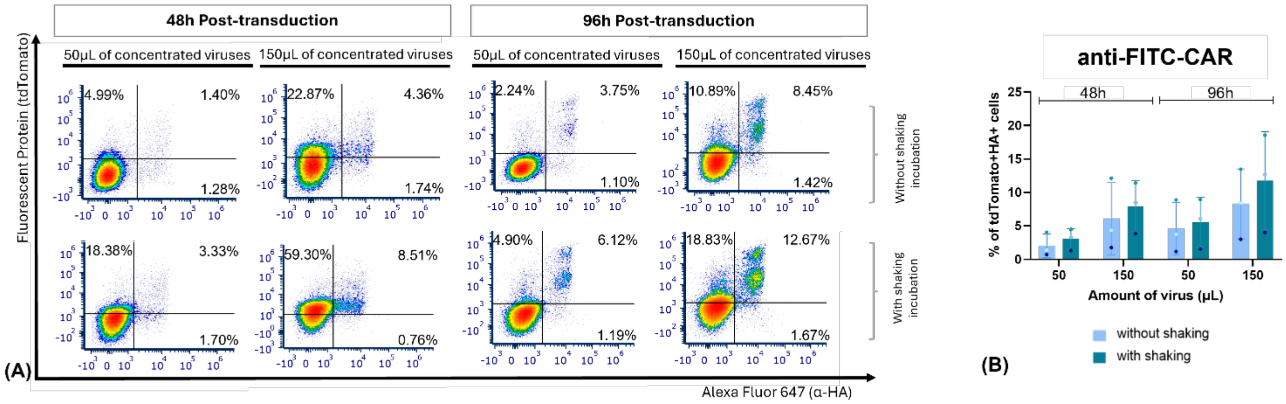

Supplementary Figure 6: Shaking incubation improved transduction. Jurkat E6-1 cells were transduced with 50µL and 150µL of anti-FITC-CAR viral particles, under shaking for 2h. Non-transduced (NT) cells were used as a negative control. Levels of tdTomato and HA tags were measured 48h and 96h post-transduction to assess the percentage of transduced cells with anti-FITC-CAR. (A) Representative plots at 48h and 96h post-transduction. (B) Data shown as mean ± SD (n=3).

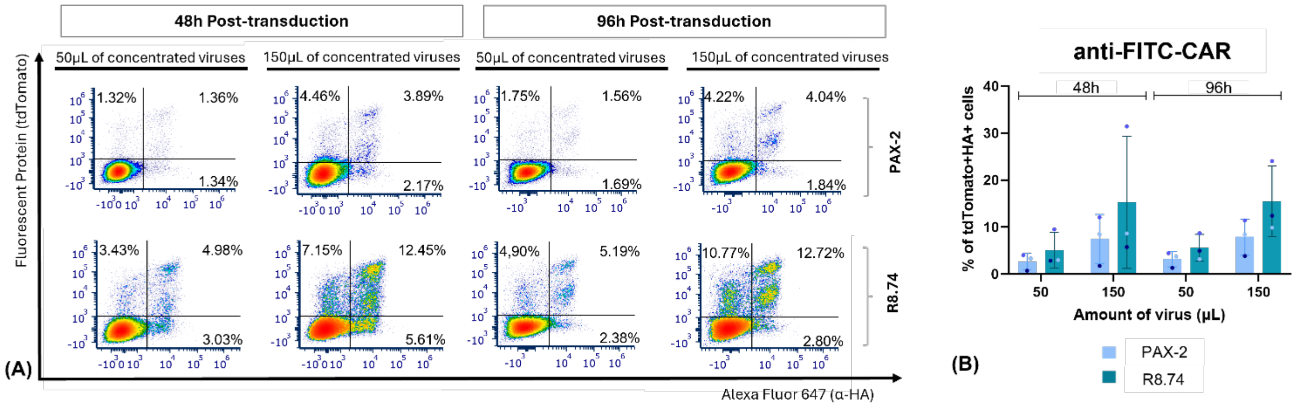

Supplementary Figure 7: R8.74 improved transduction. Jurkat E6-1 cells were transduced with 50 µL and 150 µL of anti-FITC-CAR viral particles, under shaking for 2h. LVs were generated using PAX2 or R8.74 as packaging plasmids. Non-transduced (NT) cells were used as a negative control. Levels of tdTomato and HA tag were measured 48h and 96h post-transduction to assess the percentage of transduced cells with anti-FITC-CAR. (A) Representative plots 48h and 96h post-transduction. B) Data shown as mean ± SD (n=3).

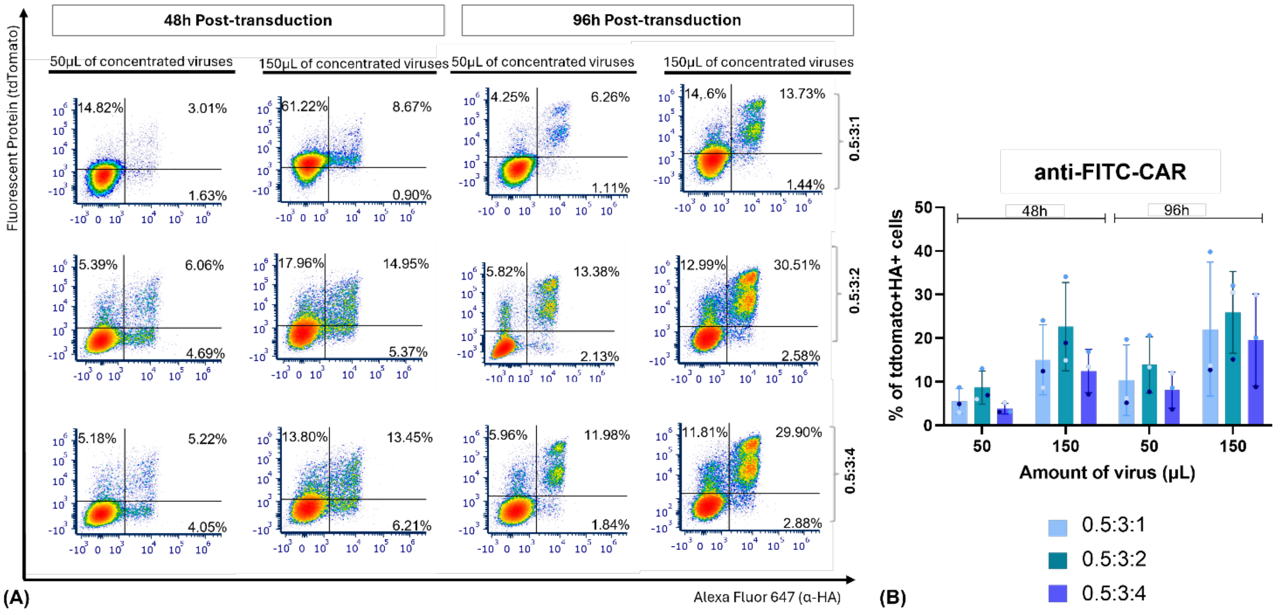

Supplementary Figure 8: 0.5:3:2 transfection ratio improved transduction. Jurkat E6-1 cells were transduced with 50µL and 150µL of anti-FITC-CAR viral particles, under shaking for 2h. LVs were made using R8.74 as packaging plasmid, and different plasmid ratios were tested (0.5:3:1, 0.5:3:2, and 0.5:3:4). Non-transduced (NT) cells were used as a negative control. Levels of tdTomato and HA tag were measured 48h and 96h post-transduction to assess the percentage of transduced cells with anti-FITC-CAR. (A) Representative plots after 48h and 96h post-transduction. (B) Data shown as mean ± SD (n=3).

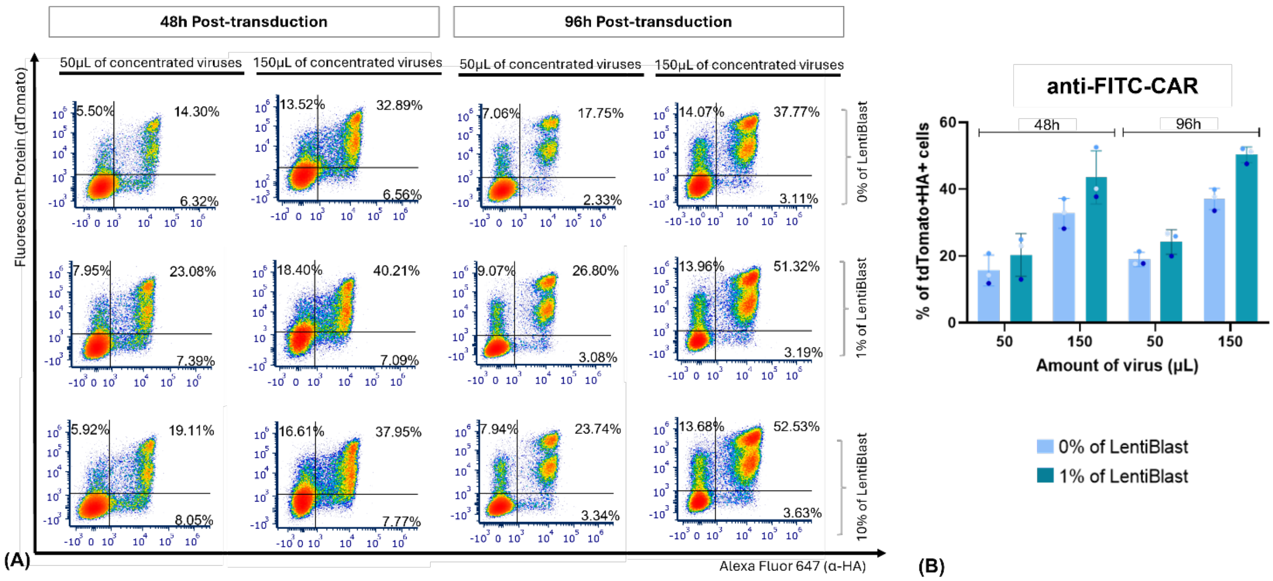

Supplementary Figure 9: LentiBlast Premium improved transduction. Jurkat E6-1 cells were transduced with 50µL and 150µL of anti-FITC-CAR viral particles and LentiBlast, under shaking for 2h. LVs were prepared using the R8.74 packaging plasmid with a 0.5:3:2 transfection ratio. Non-transduced (NT) cells were used as a negative control. Levels of tdTomato and HA tag were measured 48h and 96h post-transduction to assess the percentage of transduced cells with anti-FITC-CAR. (A) Representative plots after 48h and 96h post-transduction. (B) Data shown as mean ± SD (n=3).

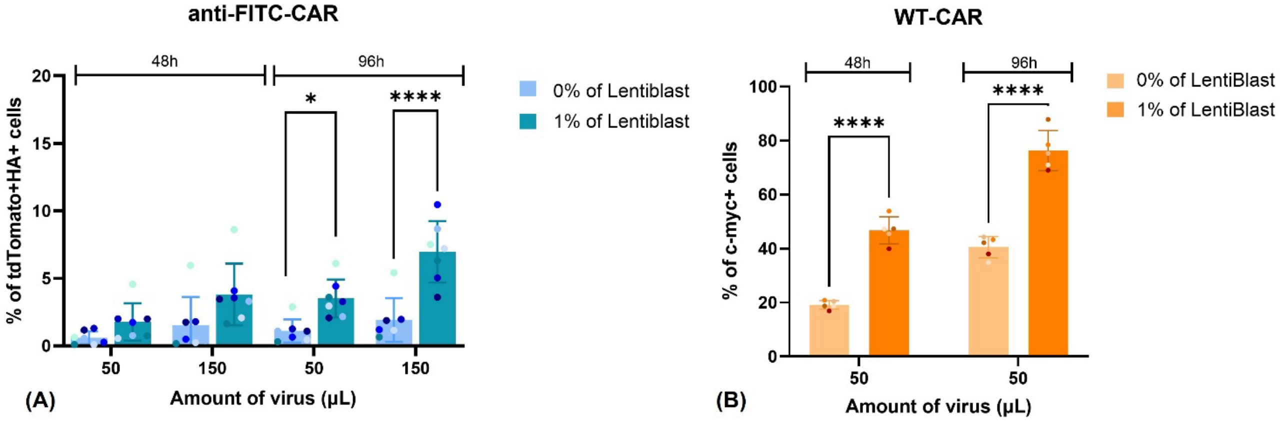

Supplementary Figure 10: Transduction of PBMCs was achieved. PBMCs were transduced with 50µL and 150µL of anti-FITC-CAR or WT-CAR viral particles of concentrated or non-concentrated LV particles, respectively. The shaking step for the first 2 h after transduction was performed using the enhancer LentiBlast. LVs were produced with R8.74 as packaging plasmid with 0.5:3:2 transfection ratio. Non-transduced (NT) cells were used as a negative control. Levels of tdTomato and HA tag (A) or c-myc tag (B) were measured 48h and 96h post-transduction to assess the percentage of transduced cells with anti-FITC-CAR or WT-CAR, respectively. Data shown as mean ± SD (n=7 for anti-FITC-CAR, n=5 for WT-CAR; *p<0.05, ****p<0.001).

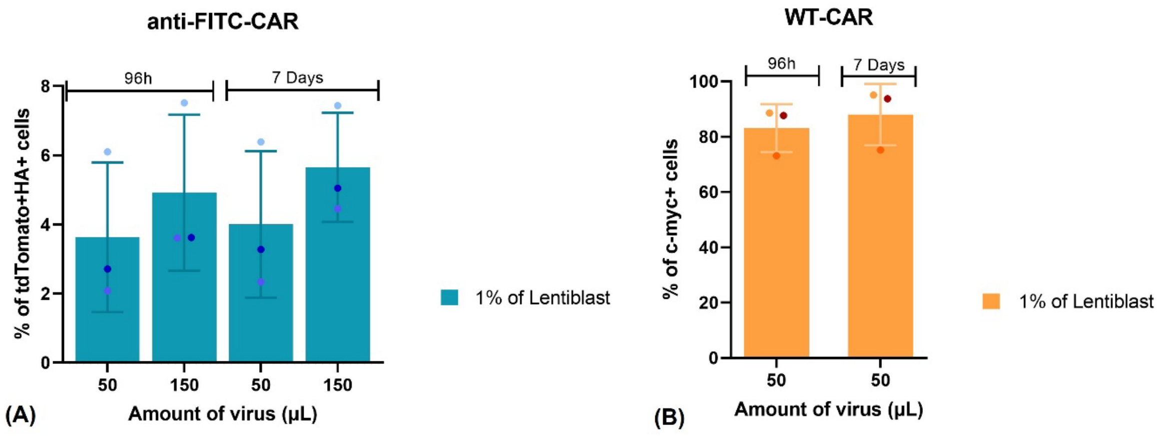

Supplementary Figure 11: Transduction is stable for 7 days. PBMCs were transduced with 50µL or 150µL of anti-FITC-CAR concentrated particles or 50µL of non-concentrated WT-CAR viral particles. The shaking step for the first 2 h after transduction was performed using the enhancer LentiBlast. LVs were produced with R8.74 as packaging plasmid with 0.5:3:2 transfection ratio. Non-transduced (NT) cells were used as a negative control. Levels of tdTomato and HA tag (A) or c-myc tag (B) were measured 96h and 7 days post-transduction to assess the percentage of transduced cells with anti-FITC-CAR or WT-CAR, respectively. Data shown as mean ± SD (n=3).

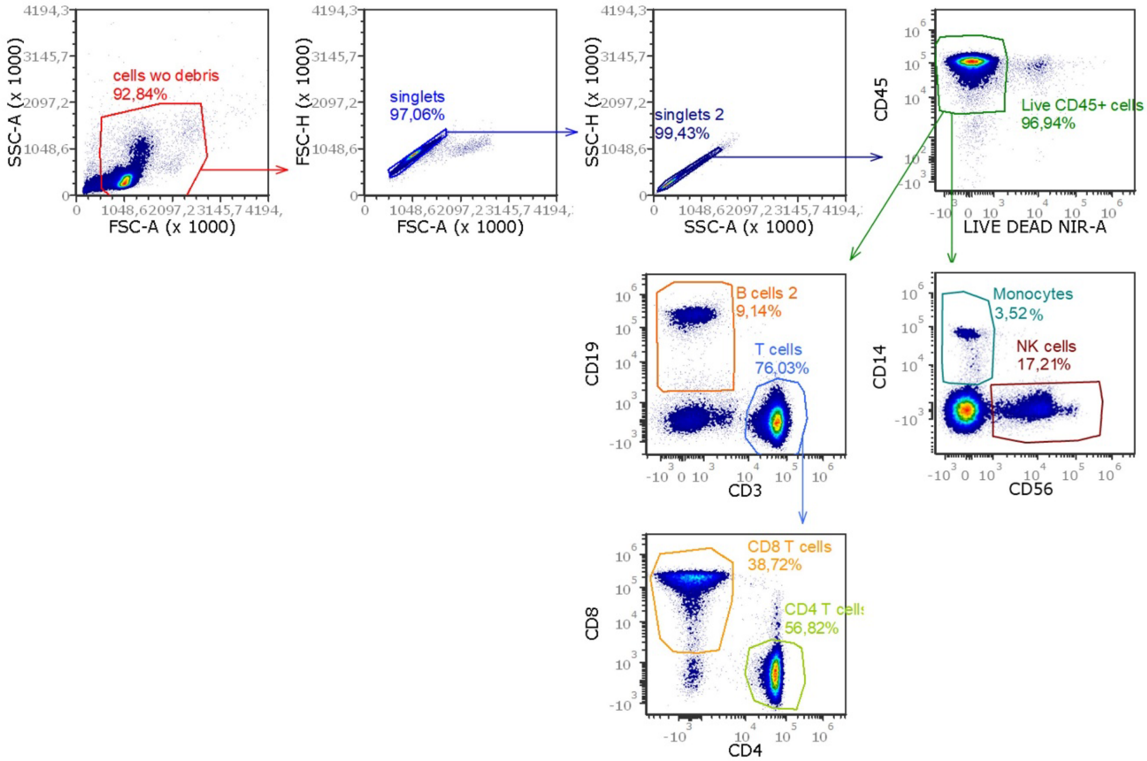

(A)

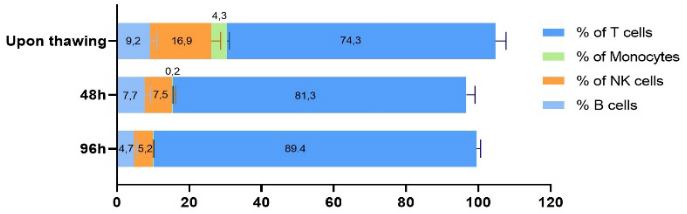

(B)

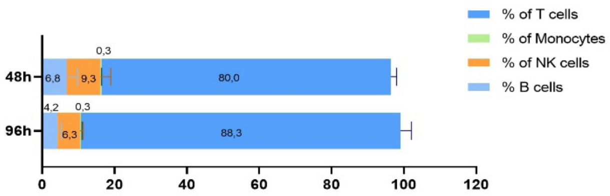

(C)

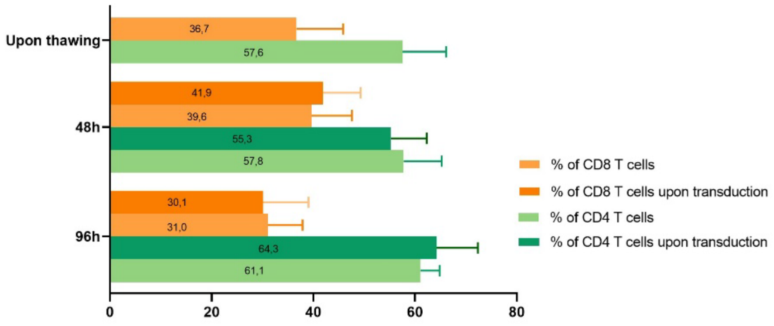

(D)

Supplementary Figure 12: Immunophenotyping of PBMCs. PBMCs were thawed for transduction assays and major populations were characterized after thawing and the days corresponding to 48h and 96h post-transduction. (A) Gating strategy is depicted. Major populations throughout the assay in non-transduced (NT) cells (B) and transduced cells (C). (D) CD4 and CD8 T cell frequencies for NT and transduced conditions. Data shown as mean ± SD (n=5 for NT, n=3 for transduced conditions).
